# Magnetic Resonance Imaging to Assess Blood–Brain Barrier Damage in Murine Trypanosomiasis

**DOI:** 10.4269/ajtmh.2011.10-0487

**Published:** 2011-02-04

**Authors:** Jean Rodgers, Christopher McCabe, George Gettinby, Barbara Bradley, Barrie Condon, Peter G. E. Kennedy

**Affiliations:** Institute of Infection, Immunity and Inflammation, College of Medical, Veterinary and Life Sciences, University of Glasgow, Glasgow, United Kingdom; Institute of Neuroscience and Psychology, Glasgow Experimental Magnetic Resonance Imaging Centre, University of Glasgow, Glasgow, United Kingdom; Department of Mathematics and Statistics, University of Strathclyde, Glasgow, United Kingdom; Magnetic Resonance Imaging Unit, Institute of Neurological Sciences, Southern General Hospital, Glasgow, United Kingdom

## Abstract

The ability of trypanosomes to invade the brain and induce an inflammatory reaction is well-recognized. This study uses magnetic resonance imaging (MRI) in conjunction with a murine model of central nervous system (CNS) stage trypanosomiasis to investigate this phenomenon at the level of the blood–brain barrier (BBB). Mice were scanned before and after administration of the contrast agent. Signal enhancement maps were generated, and the percentage signal change was calculated. The severity of the neuroinflammation was also assessed. Statistical analysis of the signal change data revealed a significantly (*P* = 0.028) higher signal enhancement in mice at 28 days post-infection (least squares mean = 26.709) compared with uninfected animals (6.298), indicating the presence of BBB impairment. Leukocytes were found in the meninges and perivascular space of some blood vessels in the infected mice. This study shows that the integrity of the BBB is compromised during CNS stage trypanosomiasis and that the impairment does not correlate with inflammatory cell infiltration.

## Introduction

Human African trypanosomiasis, or sleeping sickness, is a parasitic disease caused by infection with *Trypanosoma brucei rhodesiense* (*T. b. rhodesiense*) or *T. b. gambiense*. The parasites are transmitted through the bite of the tsetse fly insect vector. Although the two infections are geographically and clinically distinct, both forms of the disease are invariably fatal if not diagnosed and treated effectively with the appropriate drugs.^1^ *T. b. gambiense* is found in West Africa, and the infection follows a chronic course that can last for several years before death ensues. *T. b. rhodesiense* is found in East Africa and follows a more acute pattern of infection lasting only weeks to months.^2^ After infection, the disease progresses in two stages: the early or hemolymphatic stage, where the parasites proliferate in the blood, lymph, and peripheral tissues, and the late or encephalitic stage, where the trypanosomes invade and become established within the central nervous system (CNS).^2^,^3^

The pathological changes that occur during the CNS stage of the disease have been described in only a very limited number of samples. The inflammatory pattern is associated with the progressive development of a meningoencephalitis characterized by astrocyte and microglial cell activation with accompanying T-cell and monocyte transmigration into the brain. This reaction increases in severity as the disease advances, and plasma cells become a common feature within the inflammatory lesions together with the occasional Mott or Morular cell. In some instances, the reaction can take the form of an acute hemorrhagic leukoencephalopathy. However, neuronal damage and demyelination are minimal.^4^–^7^ This inflammatory pattern has been mirrored in both rodent and primate models of the human disease.^8^–^11^

Despite our knowledge of the pathological substrates of the neuroinflammatory reaction, the mechanisms that facilitate trypanosome invasion of the CNS and the effects of this process on the blood–brain barrier (BBB) remain unclear.^1^ Histopathological studies in animal models to determine the condition of the BBB after infection have produced equivocal results. A study by Philip and others^12^ showed that fluorescent dye, injected into the jugular vein, could penetrate the BBB and permeate the brain parenchyma of trypanosome-infected rats, and indicated a progressive loss of barrier integrity as the disease advanced. However, a second investigation in a rat model showed no loss of the tight junction proteins occludin or zona occludens-1 and could not detect significant albumin leakage into the brain after trypanosome infection.^13^ This suggests that trypanosomes enter the brain by a mechanism that does not result in indiscriminate damage to the BBB.

In the case of human infections, there is a paucity of data regarding the changes that occur at the BBB after infection. This is largely because of the lack of neuroimaging facilities in the countries affected by the disease. There are, however, a few reports where magnetic resonance imaging (MRI) technology has been applied. Several studies report increases in signal intensity in the internal and external capsule, corpus callosum, basal ganglia, and cerebellum after gadolinium enhancement.^14^–^17^ Because contrast agents should not readily pass through the intact BBB, these findings suggest that the integrity of the barrier has been compromised in these individuals at the time of the scan.

MRI has several advantages over the conventional histological approaches in studies investigating the BBB. The technique allows imaging of the whole brain and can indicate specific areas where changes in barrier integrity occur. Furthermore, because MRI is performed *in vivo*, any alterations to the BBB must be regarded as a consequence of the disease process and cannot be attributed to artefact as a result of the animal's death. In this investigation, we have, for the first time, applied small-bore MRI in conjunction with the administration of a gadolinium-based contrast agent to visualize and quantify changes in the BBB integrity associated with the early CNS stage of the disease using a well-established murine model of human African trypanosomiasis.

## Materials and Methods

### Animals and infections.

Female CD-1 mice (Charles River Laboratories Inc., Margate, Kent, UK) were infected with 2 × 10^4^ *T. b. brucei* parasites of cloned stabilate GVR35 by intraperitoneal injection. In this model, the mice develop a fluctuating parasitemia, and the parasites become established within the CNS between 14 and 21 days post-infection. After this time point, the infection can no longer be cured using primary stage drugs. This indicates that the disease has entered the secondary or CNS stage of the infection. The severity of the neuropathological response in these mice increases in a stepwise manner as the infection progresses.^18^ Without chemotherapeutic intervention, the animals survive for around 35 days. For the purposes of this study, three animals were prepared for MRI on day 28 post-infection. An additional six mice were killed, and their brains were excised, fixed in 4% neutral buffered formalin, and paraffin wax-processed for histological analysis. Uninfected mice were also examined in an identical fashion to the infected animals.

All animal experiments were authorized in the United Kingdom under the Animals (Scientific Procedures) Act 1986 and approved by the University of Glasgow Ethical Review Committee.

### MRI measurements.

Mice were anaesthetized with 1–2% isofluorane (Baxter Healthcare, Newbury, Berkshire, UK) delivered in a mixture of 70:30 N_2_O:O_2_. To facilitate administration of contrast agent during the scanning protocol, a tail vein was cannulated with a 26-gauge × 19-mm cannula, and the animals were placed prone into a mouse cradle. To restrict movement, the head was restrained using ear and tooth bars, and the surface coil was placed above the head of the animal. Anesthesia was maintained throughout the procedures, and respiration and heart rate were observed. Body temperature was continuously monitored by a rectal thermocouple, and the animal was maintained normothermic by an enclosed warm-water circuit.

MRI was performed on a Bruker Biospec 7T/30-cm system (Bruker, Ettlingen, Germany) equipped with an inserted gradient coil (121 mm ID, 400 mT/m) and a 72-mm birdcage resonator. A surface coil was used for brain imaging. MRI was carried out on control (non-infected mice) and 28-day post-infection mice. The protocol consisted of a rapid acquisition with relaxation enhancement (RARE) T_2_-weighted scan (effective echo time [TE] = 76 ms, repetition time [TR] = 5,362 ms, 25 averages, matrix = 176 × 176, field of view [FOV] = 17.6 × 17.6 mm, 25 contiguous coronal slices of 0.4-mm thickness) followed by a RARE T_1_-weighted scan (effective TE = 9 ms, TR = 8,000 ms, 20 averages, matrix = 176 × 176, FOV = 17.6 × 17.6 mm, 25 contiguous coronal slices of 0.4-mm thickness). After this scan, 0.1 mL of a solution containing 50 μL gadolinium-diethylenetriamine penta-acetic acid (Gd-DPTA; Magnevist, Bayer HealthCare, Uxbridge, Middlesex, UK) and 50 μL of sterile water were injected through the tail vein cannula, and the T_1_-weighted scan was repeated after a delay of 5 min after contrast agent injection. Gd-DTPA cannot readily cross the intact BBB because of its charge and high molecular weight.^19^ When injected intravenously, Gd-DTPA will accumulate in regions of damage and result in a local increase in the MRI signal intensity. Extravasation of Gd-DTPA observed within the parenchyma shows an impairment of the BBB integrity.

Image J software (http://rsbweb.nih.gov/ij/) was used for image analysis. Contrast agent enhancement maps were generated from the signal of the pre- and post-contrast T_1_-weighted scans according to the following equation*Enh* = (Spost − Spre)/Sprewhere *S_post_* is the post-contrast agent signal and *S_pre_* is the pre-contrast agent signal.

Regions of interest (ROIs) were manually defined to include the entire brain and meninges. The mean percentage signal change for each brain slice was then calculated.

### Assessing the severity of the neuroinflammation.

Hematoxylin and eosin (H&E)-stained sections through the hippocampal brain region were examined by two independent assessors in a blinded fashion. The severity of the neurological reaction was graded using a previously described grading scale (Table 1),^20^ where 0 represents a normal brain, grade 1 shows mild meningitis, grade 2 shows moderate meningitis with the development of some perivascular cuffs, grade 3 shows more severe meningitis and perivascular cuffing with the presence of some inflammatory cells in the neuropil, and grade 4 is characterized by a severe meningoencephalitis with inflammatory cells throughout the parenchyma.

### Statistical analysis.

Statistical analyses of the neuropathological response were performed. A Wilcoxon signed rank test was used to compare gradings from the two assessors for evidence of any significant difference. In addition, Fleiss' κ statistic was calculated as a measure of agreement between assessors. Thereafter, a general linear model was performed on the mean grading scores of the two assessors to test for significant differences (5% level) between the two groups of mice and to obtain an estimate of the mean difference and its 95% confidence interval.

Analysis of the post-contrast signal enhancement data from the MRI investigations was performed using a three-factor nested general linear model to test for a difference (5% level) between infected and uninfected animals and to estimate the size of the treatment effect and its 95% confidence interval. The design took account of mice being nested within treatment groups, and both mouse and slice factors were considered to be random.

## Results

### MRI.

**Figure 1.**
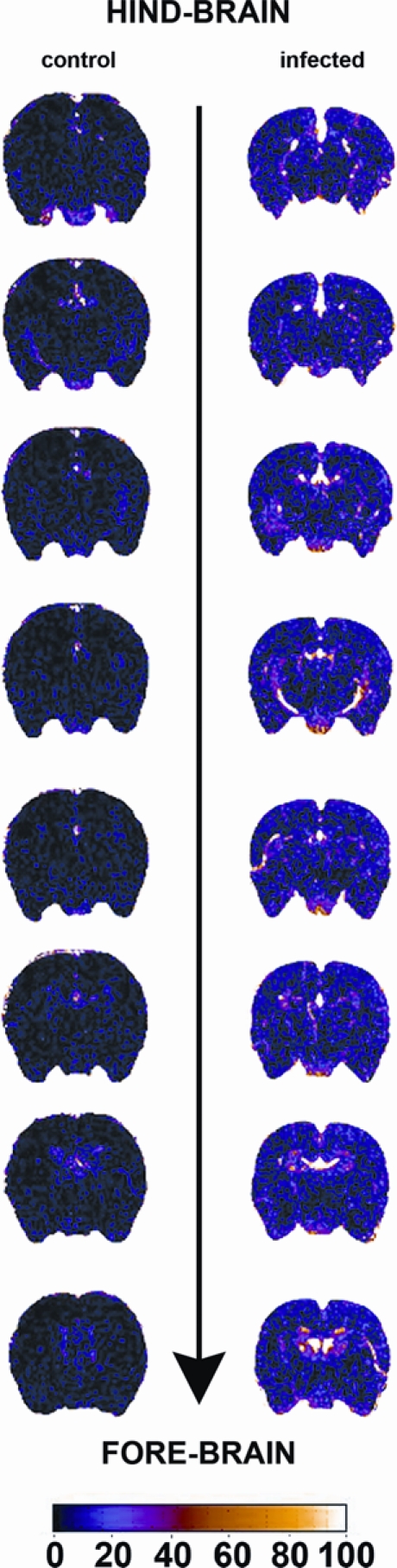
Percentage signal change map showing eight representative sections taken from a control uninfected mouse (Left) and a mouse scanned at 28 days after infection with *T. b. brucei* (Right). The color bar illustrates the percentage signal change, with brighter colors indicating higher levels of change. The areas showing the brightest colors correspond to the regions with the highest BBB impairment. These are situated in the ventricular regions; however, widespread increases in signal change over those seen in the control mouse are apparent throughout the brain sections.

**Figure 2.**
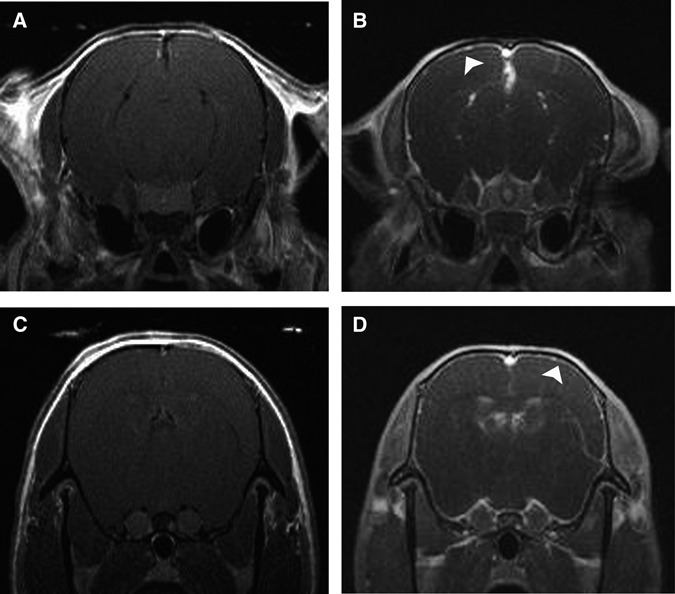
MRI scans generated after administration of contrast agent in an uninfected mouse (**A** and **C**) and an animal scanned 28 days after infection with *T. b. brucei* (**B** and **D**). Arrowheads indicate the presence of clear meningeal enhancement in the infected mouse compared with the uninfected animal. **A** and **B** correspond with the hindmost section, whereas **C** and **D** correspond with the foremost section shown in Figure 1.

The percentage signal change detected after injection of contrast agent in mice at 28 days post-infection (least squares mean = 26.709) was significantly higher (*P* = 0.028) than that seen in uninfected control animals (least squares mean = 6.298). This represented a mean increase of 20.41%, with a 95% confidence interval of 18.77–22.05. The difference between the levels of contrast agent reaching the brain in infected animals compared with their uninfected counterparts is apparent on examination of the percentage signal change maps shown in Figure 1, where images from infected mice show many areas of brighter coloration, indicating regions where contrast agent is present. In the infected animals, increased signal was identified in multiple brain regions, although the areas showing the highest level of signal change were found in the meninges and ventricular region (Figures 1 and 2). The presence of meningeal enhancement in the infected mice can be clearly visualized in the post-contrast scan images shown in Figure 2. Areas of signal change were also present in the cortex and thalamus as well as circumventricular regions, including the hypothalamus, suprachiasmatic nucleus, and median eminence. In addition, several vessels showing high levels of contrast could be recognized penetrating the cortex. In comparison, uninfected control mice showed only very limited signal enhancement that was largely confined to the ventricular area or the occasional large vessel entering the brain (Figure 1).

### Neuropathological response.

The median of the difference between the gradings of the assessors was 0, with a 95% confidence interval of 0–0.25, and this was not significant. The measure of agreement between assessors led to a κ value of 0.812, which indicated almost perfect agreement.

**Figure 3.**
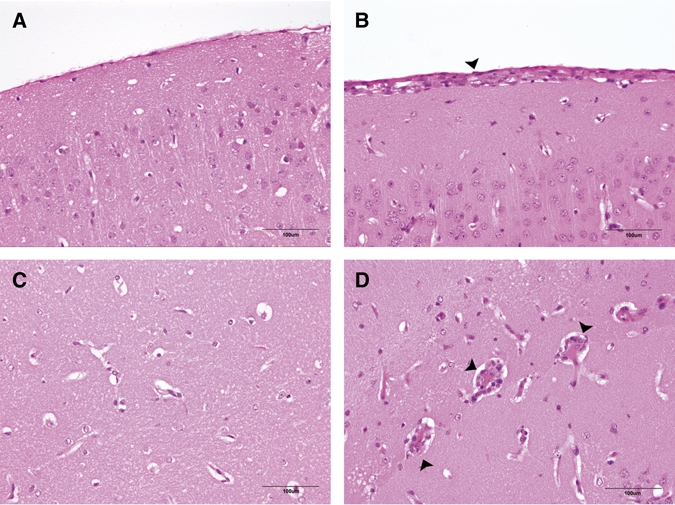
Coronal sections through the brain of a normal mouse (**A** and **C**) and a mouse killed at 28 days after *T. b. brucei* infection (**B** and **D**). Infiltrating inflammatory cells (arrowheads) can be seen in the meninges of the cerebral cortex (**B**) and surrounding the blood vessels in the hippocampal fissure (**D**) in the infected animal, whereas no inflammatory infiltrate is apparent in comparative areas in the normal mouse (**A** and **C**).

The severity of the neuropathological reaction in uninfected mice and animals at 28 days after infection was examined to determine whether the areas of signal change detected by MRI corresponded with areas of more severe inflammation in the mice. Mice killed at day 28 post-infection during the early CNS stage of the disease exhibited minor neuroinflammatory changes, with a mean neuropathology score of 1.917 ± 0.167 (mean ± standard error [SE]). This was characterized by the presence of a mild to moderate meningitis in these mice with inflammatory cells apparent in the meninges and the perivascular space surrounding some blood vessels (Figure 3B and D). The perivascular cuffing was particularly prominent around the blood vessels of the hippocampal fissure (Figure 3D). The inflammatory cell infiltrate was composed of macrophages, lymphocytes, and plasma cells. The neuropathological reaction found in mice exhibiting the early CNS stage of the disease was, as expected, significantly higher (*P* < 0.0001; 95% confidence interval for the difference in mean score = 1.545–2.288) than that seen in normal uninfected animals where no neuroinflammatory changes were detected (Figure 3A and C).

## Discussion

To date, the effects of trypanosome infection on the BBB have remained unclear. To our knowledge, this is the first report of the use of small-bore MRI to assess BBB breakdown in experimental trypanosomiasis infection. This study clearly shows that trypanosome infection compromises the integrity of the barrier when the mice exhibit pathological changes mirroring the early CNS stage of human disease. At this time, only mild to moderate inflammatory changes were detected on histological examination of brain sections, with limited inflammatory cell infiltration of the meninges and the perivascular space in some vessels. Trypanosomes were not visualized within the brain parenchyma in this study; however, previous investigations have shown numerous parasites in the interventricular foramen,^21^ and Taqman polymerase chain reaction (PCR) confirms that a substantial number of parasites are present in the brain at this point in the infection (Rodgers J and others, unpublished data). Under physiological conditions, the traffic of lymphocytes across the BBB is strictly controlled, with only a few activated cells transmigrating across the barrier.^22^ In this case, it is interesting to note the apparent disparity between the diffuse distribution and degree of BBB leakage seen on MRI examination and the localized neuroinflammatory changes exhibited by mice at this stage in the infection. This suggests that, although BBB integrity has been compromised at this point in the development of the CNS inflammatory reaction, the brain, to a large extent, remains compartmentalized from the peripheral systems and maintains the tight regulatory process responsible for controlling inflammatory cell transmigration into the brain. A similar picture has been reported in multiple sclerosis (MS) and experimental autoimmune encephalomyelitis (EAE); lymphocyte recruitment into the CNS remains tightly managed under inflammatory conditions, despite the presence of BBB impairment, because the T cells infiltrating the brain parenchyma have been shown to be phenotypically distinct from those present in other tissues.^22^ The development of leaky vessels, attributed to a loss of the tight junction protein claudin-3, that was not dependant on the presence of inflammatory cells has also been reported in glioblastoma multiforme.^23^ It is possible that the disparity seen after trypanosome infection could indicate that the BBB impairment found in animals at this stage in the disease process is not solely the result of damage caused by the ongoing inflammatory process present within the CNS compartment and may be a direct consequence of the presence of the parasite. Indeed, *T. b. rhodesiense* parasites have been shown to cross *in vitro* BBB systems using cultures of human brain microvascular endothelial cells through a paracellular route, with only a transient reduction in the transendothelial electrical resistance and causing no permanent damage to barrier integrity.^24^ Furthermore, it seems that parasite-derived cysteine protease enzymes are important factors enabling this traversal of the barrier.^25^

In MS, MRI has shown the presence of lowered BBB integrity preceding the development of clinical symptoms of the disease, and this was associated with a substantial infiltration of T cells and the presence of demyelinative lesions.^26^,^27^ Recent investigations have provided additional insight into the development of these inflammatory lesions in EAE, regarded as an animal model of MS.^28^–^30^ Using intravital two-photon microscopy, Bartholomaus and others^28^ showed that transferred myelin basic protein reactive effector T cells first appear in the subarachnoid areas, where they crawl along the inner vascular surfaces of the pial blood vessels in the spinal cord and migrate through the vessel wall to enter the subarachnoid space. Here, the T cells monitor the abluminal surface, and after an encounter with their specific antigen, presented by resident phagocytic cells in an major histocompatibility complex (MHC) II-restricted manner, they are restimulated and produce proinflammatory mediators that augment the inflammatory process including interferon (IFN)-γ and tumor necrosis factor (TNF)-α.^28^ Previous studies using a murine model of CNS human African trypanosomiasis (HAT) have shown significant increases in the levels of these two cytokines in the brain compared with uninfected control mice at 28 days post-infection.^18^ Furthermore, rising CNS concentrations of IFN-γ and TNF-α were found to correlate with an increased severity in the neuroinflammatory reaction in these mice, and increased levels of IFN-γ have been detected in the plasma^31^ and cerebrospinal fluid^32^ of patients infected with *T. b. rhodesiense* and *T. b. gambiense*, respectively. The importance of IFN-γ in the passage of trypanosomes across the BBB has been suggested by studies using IFN-γ or IFN-γ receptor knockout mice, where transmigration of trypanosomes from the cerebral blood vessels to the brain parenchyma was prevented. In addition, this impedance of parasite entry into the brain was also seen in recombination activating gene (RAG)-1 knockout mice, suggesting that T and/or B cells play a role in the transmigratory process.^33^

Several pathways have been shown to play important roles in the generation of the neuroinflammatory reaction. Studies employing substance P receptor antagonists in experimental trypanosome infections in mice have shown that this neuropeptide exerts a proinflammatory action in the brain of infected animals.^20^,^34^ Increased concentrations of this neuropeptide have also been detected in cerebral *Toxocara canis* infections in mice that, together with raised claudin-5 and neurokinin-1 receptor expression, were associated with persistent BBB impairment but little accompanying inflammation.^35^ More recently, inhibition of kynurenine-3-monooxygenase (KMO), a key enzyme in the kynurenine pathway, reduced the severity of the CNS inflammation during the late CNS stage of the disease in an experimental murine model of HAT.^21^ Furthermore, the kynurenine pathway metabolite quinolinic acid has been shown to increase the permeability of the BBB in rats to plasma albumin.^36^ Therefore, if KMO inhibition produced a reduction in quinolinic acid, this could have helped to prevent the BBB impairment associated with the trypanosome infection.

The results gained from this investigation and the investigations of Philip and others^12^ suggest that trypanosome infection reduces the integrity of the BBB.^12^ The deleterious effects of this disease on the integrity of the BBB have also been suggested by studies using *in situ* perfusion to monitor the ability of trypanocidal drugs to penetrate the CNS.^37^,^38^ In these studies, influx of both pentamidine and eflornithine into the brain parenchyma were minimal during the early stages of infection. However, the concentration of these drugs reaching the brain increased during the late stages of the disease, indicating a reduction in BBB function. These findings are in apparent contrast to those of Mulenga and others,^13^ where no changes in the distribution of occludin or zona occludens (ZO)-1 could be detected after trypanosome infection. However, it has been suggested that BBB leakiness in EAE is mainly because of opening of the tight junctions, with selective loss of claudin-3 from vessels surrounded by inflammatory cells but no apparent change in claudin-5, occludin, or ZO-1.^22^ This could help to reconcile the apparently disparate results found between the investigation by Mulenga and others^13^ and other contemporary studies examining the effects of trypanosome infection on the BBB.

We have now shown in this study that, using MRI methodology, significant BBB dysfunction can be detected in mice exhibiting the early CNS stage of the disease when mild to moderate histopathological changes are apparent. However, further detailed studies using MRI will be required to delineate and quantify the changes in BBB integrity throughout the course of trypanosome infection from the early acute stage of the disease, where no changes are detected on histopathological examination, to the post-treatment reactive encephalopathy, where the animals exhibit a severe meningoencephalitis. Although these *in vivo* studies in experimental mice have certainly provided important insights into the neuropathogenesis of human African trypanosomiasis, there is now a pressing need to use this type of neuroimaging technology wherever feasible in human patients with sleeping sickness. This will be important not only to improve our understanding of how the parasite gains access to the CNS to produce neurological disease but also to facilitate the development and in due course, assessment of new drug therapies.

Our findings in this model also have clear diagnostic implications for sleeping sickness in humans. According to the widely used World Health Organization criteria,^39^ a CSF pleocytosis of ≥ 5 white blood cells/μL is taken as a key diagnostic marker of late-stage disease, indicating that CNS involvement has occurred after BBB breakdown. However, our results reported here in mice suggest that such a close correlation between early BBB breakdown and brain involvement may not be as close or justified as generally thought. If so, then this further highlights the very difficult problem of diagnostic markers for CNS trypanosomiasis, one that is particularly acute given the exceptional toxicity of current drug therapy for late-stage sleeping sickness.^2^

## Figures and Tables

**Table 1:** Outline of the parameters used to define the neuropathological injury score allocated to grade the severity of the neuroinflammatory reaction encountered in the murine CNS after trypanosome infection^20^

	Score				
	0	1	2	3	4
Meningitis	None	Mild	Moderate	Severe	Severe
Perivascular cuffing	None	None	Mild cuffing of some vessels	Prominent cuffing of some vessels	Prominent cuffing of most vessels
Encephalitis as defined by cellular activity in the neuropil	None	None	None	Moderate	Severe
